# Evaluating Disease Threats to Sustainable Poultry Production in Africa: Newcastle Disease, Infectious Bursal Disease, and Avian Infectious Bronchitis in Commercial Poultry Flocks in Kano and Oyo States, Nigeria

**DOI:** 10.3389/fvets.2021.730159

**Published:** 2021-09-08

**Authors:** Abel B. Ekiri, Bryony Armson, Kehinde Adebowale, Isabella Endacott, Erika Galipo, Ruth Alafiatayo, Daniel L. Horton, Adah Ogwuche, Olorunsola N. Bankole, Hussein M. Galal, Beatty-Viv Maikai, Mariana Dineva, Aliyu Wakawa, Erik Mijten, Gabriel Varga, Alasdair J. C. Cook

**Affiliations:** ^1^Department of Veterinary Epidemiology and Public Health, School of Veterinary Medicine, University of Surrey, Surrey, United Kingdom; ^2^Zoetis-ALPHA Initiative, Zaventem, Belgium; ^3^Department of Microbiology, Faculty of Veterinary Medicine, Cairo University, Giza, Egypt; ^4^Department of Veterinary Public Health and Preventative Medicine, Ahmadu Bello University, Zaria, Nigeria; ^5^Department of Veterinary Medicine, Ahmadu Bello University, Zaria, Nigeria

**Keywords:** Newcastle disease, infectious bursal disease, avian infectious bronchitis, virus, poultry, chicken, Nigeria, Africa

## Abstract

The growth of the poultry industry in Nigeria is constrained by major poultry diseases, despite the implementation of vaccination programs. This study aimed to assess the level of protection against Newcastle disease (ND), infectious bursal disease (IBD), and avian infectious bronchitis (IB) afforded by current vaccination schedules and characterize the circulating virus strains in commercial poultry flocks in Nigeria. A cross-sectional study was conducted on 44 commercial poultry farms in Oyo and Kano states of Nigeria. Serum and tissue samples and data on flock, clinical and vaccination records were collected on each farm. Farms were classified as being protected or not protected against ND, IBD and IB based on a defined criterion. Real-time reverse transcription polymerase chain reaction (rRT-PCR) testing was performed for each target virus on tissue samples and positive samples were sequenced. A total of 15/44 (34.1%), 35/44 (79.5%), and 1/44 (2.3%) farms were considered to be protected against ND, IBD, and IB, respectively, at the time of sampling. NDV RNA was detected on 7/44 (15.9%) farms and sequences obtained from 3/7 farms were characterized as the lentogenic strain. Infectious bursal disease virus (IBDV) RNA was detected on 16/44 (36.4%) farms tested; very virulent (vv) IBDV and non-virulent (nv) IBDV strains were both detected in 3/16 (18.8%) positive samples. Sequences of IBDV isolates were either clustered with a group of genotype 3 virulent IBDV strains or were related to vaccine strains MB and D78 strains. IBV RNA was detected on 36/44 (81.8%) farms, with variant02, Massachusetts, 4/91, and Q1 variants detected. Sequences of IBV isolates were either clustered with the vaccines strains Massachusetts M41 and H120 or were most closely related to the D274-like strains or a clade of sequences reported in Nigeria and Niger in 2006 and 2007. This study revealed that most study farms in Oyo and Kano states did not have adequate protective antibody titers against IBV and NDV and were therefore at risk of field challenge. Infectious bursal disease virus and IBV RNA were detected on farms with a history of vaccination suggesting potential vaccination failure, or that the vaccine strains used mismatch with the circulating strains and are therefore not protective.

## Introduction

Agriculture continues to be the most important sector of the Nigerian economy in terms of provision of employment, in spite of its declining contribution to the nation's foreign exchange earnings (1). About 65% of Nigerians are estimated to depend on agriculture for their livelihood, while 34.8% of the GDP, and over 38% of the non-oil foreign exchange earnings, are contributed by the agricultural sector (1). Within the agriculture sector, sustainable development of poultry production can make an important contribution to the UN Sustainable Development Goals, providing affordable protein and potentially mitigating climate change (1). With a population of nearly 200 million birds (2), poultry is the most commercialized agricultural livestock sector in Nigeria. Poultry production has expanded rapidly and Nigeria is now the largest poultry producer in Africa (3).

The growth of the poultry industry in Nigeria is however limited by several viral diseases including Newcastle disease (ND), infectious bursal disease (IBD), and avian infectious bronchitis (IB), which result in severe production and economic losses to poultry farmers (4–6). For the years 2009–2011, estimated economic losses experienced by poultry farmers amounted to over three billion Nigerian Naira (approximately 7.3 million US dollars) due to IBD outbreaks alone (7). Newcastle disease and IBD are the two most dreaded viral diseases of poultry in Nigeria causing illness, reduced egg production, immunosuppression, and often death, following infection with pathogenic strains of their respective causative viruses (8–11). Despite efforts to prevent and control ND and IBD, continued circulation of the causative viruses among free-roaming and wild birds has been reported as one of the factors responsible for the sporadic outbreaks of ND and IBD among free-roaming village chickens and commercial poultry flocks (12–15).

Avian IB is one of the most important viral respiratory diseases of chickens in Africa, where it is widespread in vaccinated and unvaccinated poultry farms (16, 17). A high prevalence of IB was reported in West and North Africa (18–20) and in Nigeria; 84% seroprevalence was reported in 1059 commercial chickens in the south-western part of the country in 2016 (18) and 95% in free-range chickens in Oyo (21). Despite this, little is known about the molecular and serological characteristics of the circulating viral variants, limiting the ability to design effective control programmes. In 2017, anecdotal reports from veterinarians in Nigeria indicated that an unusually high number of losses suspected to be due to IBV infection occurred in commercial poultry flocks that were vaccinated against IBV suggesting that the vaccines that are currently in use might not be effective against the circulating strains.

In order to determine whether this was true, this study aimed to identify and characterize IBV, but also NDV, and IBDV strains circulating in commercial poultry flocks in Oyo and Kano states of Nigeria, so that the information obtained may be used to inform effective vaccine selection. Additionally, it was important to assess the level of protection afforded by the routine vaccination schedule on these poultry farms, so that recommendations for improvements on individuals farms may be provided.

## Materials and Methods

The institutional ethical review and approvals were granted by the Ahmadu Bello University, Nigeria, Ethical committee (Ethics approval number: ABUCAUC/2018/055) and by the University of Surrey, United Kingdom, Animal Welfare and Ethical Review Board (NASPA Reference: NERA-1819-003).

### Study Population

The target study population was poultry flocks on farms housing chickens reared for commercial purposes, including broilers, layers, and breeders in Oyo and Kano states of Nigeria.

### Study Area

**Figure 1:**
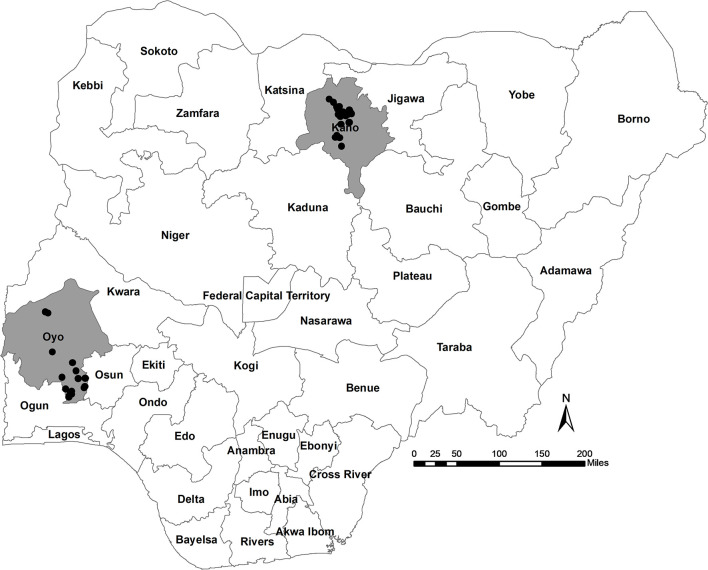
Location of participating study farms (black circles) in Oyo and Kano States, Nigeria.

The study area included two states of Nigeria; Oyo, located in the south-west; and Kano, located in north-central Nigeria (Figure 1). The two states were purposively selected based on the following criteria: (i) a high concentration of commercial poultry production, as determined from verbal communications with key opinion leaders in veterinary private practice, poultry associations, and from local poultry research experts; (ii) a high human population; (iii) variation in agro-ecological zones; (iv) logistical considerations including security status, transportation, and co-ordination of activities; and (v) laboratory access (the target laboratory for sample serology testing was located in Ibadan, south-west region).

### Study Approach

This study was part of a larger project that investigated poultry health, production and management, and veterinary pharmaceutical use amongst commercial poultry farmers in Kano and Oyo states of Nigeria (Ekiri et al., in preparation, Endacott et al., in preparation). A cross-sectional study was performed between 10/05/2019 and 08/06/2019. The selected study farms were visited, and serum samples were collected from live birds to estimate antibody titer status against NDV, IBV, and IBDV. In addition, tissue samples were collected from selected sick and dead birds to detect the presence of NDV, IBV, and IBDV RNA. Farm visit dates were determined by the study team based on logistical arrangements.

### Sampling Approach

A list of poultry farms and the contact information available in each target state was obtained from state poultry associations. A two-stage sampling method was then used to select participating study farms in each state and study birds on each selected farm. Within each state, poultry farms were randomly selected using probability-proportional-to-size sampling; the number of farms selected in each state were proportionate to the number of farms present in the state.

### Sample Size

The sample size was estimated based on a sero-prevalence of 20% for ND (22), between-cluster variance of 10%, average cluster size of 2,000 birds, and a desired confidence interval of 95% at an allowable error of 10%, as described by Levy and Lemeshow (23). Consequently, a sample size of at least 38 commercial poultry farms was estimated in the two selected states, with 18 live birds to sample per farm, resulting in a total of 684 birds. Six farms (15%) were added to the 38 farms to account for attrition, resulting in a total of 44 farms across the two states, therefore a total of 792 birds. Considering the probability-proportional-to-size sampling at state level, 16 poultry farms were randomly selected in Oyo [(estimated sample size/total number of farms in both Oyo and Kano states) × number of farms in each state: (44/112) × 40 = 16] and 28 farms in Kano [(44/112) × 72 = 28].

To determine the “study flock” on a farm, if a farm housed only one bird type (i.e., either broiler, layer, or breeder birds), then birds were randomly sampled from across the farm. However, where a farm housed multiple bird types, layer birds only were chosen, due to the greater risk of exposure related to staying longer on the farm. Subsequently, where farms contained several flocks in different houses, the data for this study only relate to the sampled flock.

Serum samples were collected from a fixed number of live birds (*n* = 18) per study flock, regardless of farm size. In addition, two apparently sick or dead birds were opportunistically sampled per study flock (2 × 44 = 88 birds) at post-mortem. Farmers were compensated at market value for any sick birds that were culled for post-mortem examination. In total, 880 birds were sampled; serology samples were collected from 792 birds, and tissues or swabs were collected from 88 birds.

### Data Collection

#### Farmer Survey

A questionnaire was administered by interview to poultry farmers on each visited farm to collect data on the study flocks, including clinical and vaccination records. Survey data were captured using the platform, Qualtrics.

#### Sample Collection

Blood samples (2 ml) were collected from the brachial and/or jugular vein of each bird using a 5 ml syringe and 23-gauge × 1.5-inch needle. The blood was transferred into vials and kept in a slanted position for at least 25 min to allow the blood to clot. Serum was then aliquoted into 2 ml tubes and transported in a cooler to the laboratory. Blood samples were collected during the study farm visit. The farm visit dates were determined by the study team based on logistical arrangements.

A post-mortem examination was conducted on the selected sick, culled or dead birds and six samples were collected per bird: cloacal, oropharyngeal and tracheal swabs, proventriculus, bursa, and caecal tonsil tissue. The following samples were used for the target pathogens: cloacal swab and proventriculus for the detection of NDV; oropharyngeal and tracheal swabs for the detection of NDV and IBV; caecal tonsil for the detection of IBV, and bursa for the detection of IBDV. For molecular testing, all relevant tissue/swab samples from two birds per farm were pooled by blotting/smearing onto one sample area of a Whatman® FTA® card (Cytiva, Global Life Sciences Solutions Operations UK Limited, UK) and transported to AniCon Laboratory (Germany) for real-time reverse transcription-polymerase chain reaction (rRT-PCR) testing and sequence analysis.

### Laboratory Testing

#### Serological Testing

The detection of antibodies from serum was conducted at Chi Lab (Ibadan, Nigeria) using the following serological tests according to the manufacturer's guidelines: IBV—ProFLOK® IBV ELISA kit (Zoetis); NDV—ProFLOK® NDV ELISA kit (Zoetis); and IBD—ProFLOK® IBD ELISA kit/ ProFLOK PLUS IBD ELISA kit (Zoetis). Serum samples were stored at 4°C, and then at room temperature (22–26°C) for 2 h prior to performing serological assays. The optical density was measured using a Biotek® ELX 800 ELISA Reader with the e-LISA 3.0.0.0 software (© Zoetis).

#### RNA Extraction

All molecular testing was performed at AniCon laboratories in Germany. For each pooled sample, a laser-cut fraction of the Whatman® FTA card was suspended in buffer for FTA-card-washout and then this was used as the sample. RNA was extracted using the Kylt® RNA/DNA Purification Kit HTP according to the manufacturer's guidelines on a customized Microlab® STAR^TM^let robot (Hamilton, UK). A beta-actin internal control was included in the extraction procedure.

#### Real-Time(r) RT-PCR

NDV, IBDV, and IBV real-time RT-PCR assays were performed on a BioRad CFX384 machine using the Kylt® Paramyxovirus 1 Real-Time RT-PCR Detection kit, Kylt® IBDV Screening Real-Time RT-PCR detection kit, Kylt® IBDV Serotype 1 Pathotyping Real-Time RT-PCR kit, and Kylt® IB-aCoV kit, respectively, according to manufacturer's instructions (Supplementary Table 1). The cycling conditions are described in the Kylt® Profile 1 (AniCon, Germany). Positive and negative controls were included in the kits. No cut-off for positivity was implemented, and therefore a positive result was defined as a *C*_*T*_-value of <42. The Kylt® IBDV Serotype 1 Pathotyping Real-Time RT-PCR kit differentiates between the very virulent (vv) and non-virulent (nv) IBDV strains. Samples tested for IBDV and IBV were first screened for the presence of IBDV and IBV RNA, and then those that tested positive were further tested with the Kylt® IBDV Serotype 1 Pathotyping Real-Time RT-PCR kit and variant-specific IBV rRT-PCR assays, respectively (Supplementary Table 1).

#### Sequence Analysis

Sanger di-deoxy sequencing was performed on those samples that tested positive for NDV, IBDV, and IBV at AniCon laboratories in Germany. For IBV, sequencing was only performed for those positive samples with a *C*_*T*_-value of ≤ 30, due to the likelihood of returning a good quality sequence. Amplification was performed using various confidential in-house PCR setups on different PCR machines. Sequences obtained were compared with available sequences for vaccine strains from the AniCon laboratory (Germany) and NCBI (https://www.ncbi.nlm.nih.gov/).

For NDV-positive samples, a 360 bp fragment of the fusion (*F*) protein coding gene of paramyxovirus-1 (PMV-1) was sequenced, and a conceptual translation was generated. The pathogenicity of the analyzed PMV-1 strains was classified based on the *F2/F1* cleavage site. For IBDV-positive samples, a 540 bp fragment of the *VP1* gene and a 550 bp fragment of the *VP2* gene were sequenced. For IBV-positive samples, a 540 bp fragment of the *S1* spike protein coding gene was sequenced.

#### Phylogenetic Analyses

Evolutionary relationships between virus sequences detected in this study and selected reference sequences were inferred for IBDV and IBV using a maximum likelihood approach implemented in MEGA-X (24). Sequences were first aligned using MUSCLE, and the Tamura-Nei evolutionary model was used with 500 bootstrap replicates. For IBDV, a 394 nucleotide region of the viral protein 2 (*VP2)* gene was used with a total of 29 sequences. For IBV a 470 nucleotide region of the S1-spike protein was used with a total of 32 sequences.

### Data Analyses

#### Serology Results Interpretation

The geometric mean (GMT) antibody titers against NDV, IBDV, and IBV and the coefficient of variation (CV) were calculated from the ELISA titers for all birds sampled (*n* = 18) in each study flock (Supplementary File 1). The following GMT antibody titer cut-offs were used for interpretation of serological results at the flock level: 2,000 for NDV live vaccine and 8,000 for NDV inactivated/killed vaccine; 2,000 for IBDV live or inactivated/killed vaccine; 6,000 for IBV classic strains live vaccine, and 15,000 for IB variant strains or classic strains inactivated/killed vaccine. These are the recommended minimum cut-off values commonly used by poultry veterinarians practicing in Ibadan, Oyo, provided by leading poultry veterinarians practicing in the area. Additionally, the proportions of birds in each flock above these recommended minimum antibody titers were also calculated for each pathogen.

Each of the 44 flocks was classified as being either “protected” or “non-protected” against ND, IBD, and IB (Supplementary File 1), based on specific parameters for each flock as recommended by the ELISA test manufacturer's instructions and on select clinically relevant factors, including the following:

i) the recommended minimum GMT antibody titers considered protective against NDV, IBDV, and IBV when using live vaccines or inactivated/killed vaccines (≤ 2000 for NDV live vaccine and ≤ 8,000 for NDV inactivated/killed vaccine; ≤ 2,000 for IBDV live or inactivated/killed vaccine; ≤ 6,000 for IBV classic strains live vaccine, and ≤ 15,000 for IB variant strains or classic strains inactivated/killed vaccine; *n* = 18 birds sampled per farm/flock);
ii) the proportion of birds in the study flock with the recommended minimum antibody titers considered protective; a proportion of ≥85% (for NDV) and 100% (for IBDV and IBV) of the sampled birds ≥ the recommended minimum antibody titer was considered acceptable flock immunity;
iii) the CV for antibody titers of the sampled birds per farm; a CV of <40% was considered good and acceptable in terms of having homogenous titers across the 18 sampled birds per flock, and a CV of >40% was considered poor and unacceptable;
iv) the vaccination records, including dates of vaccination, number of doses of NDV, IBDV, and IBV vaccines administered, type of vaccine (monovalent, bivalent), and strains present in vaccine administered;
v) the time interval between sampling date and last vaccination (for NDV, IBDV, or IBV); antibody titers attributable to the last vaccination were considered only in birds that were at least 3 weeks post vaccination;
vi) the age of birds (layer birds aged 16 weeks, or more were considered resistant to IBDV infection because the bursa would be regressing at that age).

In addition, although they were not used to determine protection, the reported flock history, including mortalities, clinical symptoms, and records of infection were considered because these factors may influence the antibody titer levels reported.

#### Statistical Analyses

Data analyses were performed using R (version 1.3.1093) (25) within the RStudio IDE (26). Pearson's chi square tests were performed to examine associations between two selected outcome variables and selected exploratory variables. The two outcome variables were: (i) protection against each pathogen (yes/no) and (ii) rRT-PCR results for each pathogen [i.e., (NDV, IBDV, or IBV) viral RNA detected on a farm: yes/no]. The explanatory variables studied were state, type of bird (broiler or layer), flock information, clinical records, vaccine records. The Venn diagram was created using Venny 2.1 (Oliveros^1^).

## Results

### Description of Study Flocks

A total of 44 farms were visited during the study period, 28 in Kano State (farm IDs 1–28) and 16 farms in Oyo State (farm IDs 29–44, Supplementary File 1). Nine of the 44 farms housed broiler birds only and 21 farms housed layer birds only. Twelve farms housed both broiler and layer birds and therefore only layer birds from these farms were considered as the study flock. The remaining two farms housed broiler-breeder birds. Most of the study flocks were layer flocks (*n* = 33, Oyo = 12/16 farms, Kano = 21/28 farms). The remaining flocks (*n* = 9) were broiler (*n* = 9, Oyo = 2, Kano = 7), or broiler-breeder chickens (*n* = 2, Oyo = 2, Kano = 0) (Table 1; Supplementary Table 2; Supplementary File 1). Where they are not specified below (section Flock Vaccination Records onwards), broiler-breeder flocks have been removed from the analysis when bird types are compared.

**Table 1:** Descriptive analysis of study flocks, evaluating associations between select flock variables and cases of NDV, IBDV, and IBV as determined by rRT-PCR.

**Variable**	**Response**	**Kano (** ***n*** **=** **28)**				**Oyo (** ***n*** **=** **16)**				**Total (** ***n*** **=** **44)**						
		**Freq**	**NDV cases (row %)**	**IBDV cases (row %)**	**IBV cases (row %)**	**Freq**	**NDV cases (row %)**	**IBDV cases (row %)**	**IBV cases (row %)**	**Freq**	**NDV cases (row %)**	**NDV pos/neg *p-*value*^a^***	**IBDV cases (row %)**	**IBDV pos/neg *p-*value*^a^***	**IBV cases (row %)**	**IBV pos/neg *p-*value*^a^***
Bird type	Broiler	7	2 (28.6)	4 (57.1)	7 (100.0)	2	1 (50.0)	1 (50.0)	1 (50.0)	9	3 (33.3)	0.250	5 (55.6)	0.347	8 (88.9)	0.622
	Layer	21	3 (14.3)	7 (33.3)	18 (85.7)	12	1 (8.3)	3 (25.0)	8 (66.7)	33	4 (12.1)		10 (30.3)		26 (78.8)	
	Broiler-breeder	0	0	0	0	2	0	1 (50.0)	2 (100.0)	2	0		1 (50.0)		2 (100.0)	
Age category^b^ (broilers)	2– <4 weeks	0	0	0	0	2	1 (50.0)	1 (50.0)	1 (50.0)	2	1 (50.0)	1.000	1 (50.0)	1.000	1 (50.0)	0.479
	4– <8 weeks	7	2 (28.6)	4 (57.1)	7 (100.0)	0	0	0	0	7	2 (28.6)		4 (57.1)		7 (100.0)	
Age category^b^ (layers)	4– <8 weeks	3	0	3 (100.0)	3 (100.0)	0	0	0	0	3	0	0.001^c^	3 (100.0)	0.004^c^	3 (100.0)	0.522^c^
	8– <20 weeks	3	2 (66.7)	3 (100.0)	3 (100.0)	1	1 (100.0)	0	1 (100.0)	4	3 (75.0)		3 (75.0)		4 (100.0)	
	20– <52 weeks	9	1 (11.1)	1 (11.1)	8 (88.9)	8	0	3 (37.5)	5 (62.5)	17	1 (5.9)		4 (23.5)		13 (76.5)	
	≥52 weeks	4	0	0	3 (75.0)	3^a^	0	0	2 (66.7)	7^a^	0		0		5 (71.4)	
	Not specified	2	0	0	1 (50.0)	0	0	0	0	2	0		0		1 (50.0)	
Flock size	<200	1	1 (100.0)	0	1 (100.0)	0	0	0	0	1	1 (100.0)	0.294	0	0.477	1 (100.0)	0.635
	200– <500	7	1 (14.3)	2 (28.6)	7 (100.0)	0	0	0	0	7	1 (14.3)		2 (28.6)		7 (100.0)	
	500– <2,000	9	2 (22.2)	4 (44.4)	8 (88.9)	1	0	0	0	10	2 (20.0)		4 (40.0)		8 (80.0)	
	2,000– <5,000	3	1 (33.3)	2 (66.7)	3 (100.0)	9	0	4 (44.4)	7 (77.8)	12	1 (8.3)		6 (50.0)		10 (83.3)	
	5,000– <15,000	7	0	2 (28.6)	5 (71.4)	6	2 (33.3)	1 (16.7)	4 (66.7)	13	2 (15.4)		3 (23.1)		9 (69.2)	
	≥15,000	1	0	1 (100.0)	1 (100.0)	0	0	0	0	1	0		1 (100.0)		1 (100.0)	
Mortality/Week	<5	14	4 (28.6)	3 (21.4)	12 (85.7)	9	1 (11.1)	2 (22.2)	5 (55.6)	23	5 (21.7)	0.145	5 (21.7)	0.030	17 (73.9)	0.534
	5–9	5	0	4 (80.0)	4 (80.0)	3	1 (33.3)	1 (33.3)	2 (66.7)	8	1 (12.5)		5 (62.5)		6 (75.0)	
	10–19	1	0	1 (100.0)	1 (100.0)	2	0	1 (50.0)	2 (100.0)	3	0		2 (66.7)		3 (100.0)	
	20–49	5	0	0	5 (100.0)	2	0	1 (50.0)	2 (100.0)	7	0		1 (14.3)		7 (100.0)	
	50–99	1	1 (100.0)	1 (100.0)	1 (100.0)	0	0	0	0	1	1 (100.0)		1 (100.0)		1 (100.0)	
	≥100	2	0	2 (100.0)	2 (100.0)	0	0	0	0	2	0		2 (100.0)		2 (100.0)	
Clinical records^d^	Drop in production	0	0	0	0	3	0	1 (33.3)	3 (100.0)	3	0	0.502	1 (33.3)	0.217	3 (100.0)	0.661
	Respiratory distress	4	1 (25.0)	2 (50.0)	4 (100.0)	0	0	0	0	4	1 (25.0)		2 (50.0)		4 (100.0)	
	Heat stress	5	0	1 (20.0)	4 (80.0)	0	0	0	0	5	0		1 (20.0)		4 (80.0)	
	Stunted growth	1	0	1 (100.0)	1 (100.0)	1	1 (100.0)	0	1 (100.0)	2	1 (50.0)		1 (50.0)		2 (100.0)	
	Diarrhea	2	1 (50.0)	2 (100.0)	2 (100.0)	2	0	2 (100.0)	2 (100.0)	4	1 (25.0)		4 (100.0)		4 (100.0)	
	Sudden deaths	0	0	0	0	2	0	1 (50.0)	2 (100.0)	2	0		1 (50.0)		2 (100.0)	
	None reported	8	1 (12.5)	2 (25.0)	7 (87.5)	4	0	1 (25.0)	2 (50.0)	12	1 (8.3)		3 (25.0)		9 (75.0)	

a *p-value according to Pearson's Chi-squared test*.

b *Broiler-breeders removed from analysis for age category*.

c *Farms that did not specify the age of their birds were not included in statistical analysis*.

d *Five farms in Oyo did not provide details of recent clinical records*.

The mean age of layer birds was 37.4 weeks [range (min–max): 6–96 weeks], of broilers was 4.8 weeks [range (min–max): 2–7 weeks], and the two broiler-breeder flocks were aged 18 and 52 weeks. Two farmers did not specify the ages of their layer flocks (Table 1; Supplementary Table 2; Supplementary File 1).

The mean layer flock size was 4,337 birds [range (min–max): 200–16,088], the mean broiler flock size was 2,467 birds [range (min–max): 100–10,000], and broiler-breeder flock sizes were 3,500 and 3,800 (Table 1; Supplementary Table 2; Supplementary File 1).

Farmers were asked whether they had experienced any recent history of bird mortality in the current production cycles. All farms apart from one (in Oyo) reported the mortality of at least 1 bird per week in the study flocks (Table 1; Supplementary Table 2; Supplementary File 1). The mean mortality per week was reported as 7.4 birds in Oyo State [range (min–max): 0–30] and 22.2 birds in Kano State [range (min–max): 1–200]. The reported mean mortality per week for layer flocks was 16.5 birds [range (min–max): 0–200], for broiler flocks was 17.2 birds [range (min–max): 2–85], and broiler-breeder flocks reported 15 and 25 mortalities per week. Accurate data was not available to enable calculation of percentage mortalities in each study flock.

Farmers were asked whether birds in the study flocks were exhibiting any clinical signs consistent with infectious disease in the current production cycle (Table 1; Supplementary Table 2; Supplementary File 1). Five farms (Oyo: 5) did not provide details of recent clinical history. For those farms that did, farmers reported a drop in production (Oyo: 3/11; Kano: 0/28), respiratory distress (Oyo: 0/11; Kano: 4/28), heat stress (Oyo: 0/11; Kano: 5/28), stunted growth (Oyo: 1/11; Kano: 1/28), diarrhea (Oyo: 2/11; Kano: 1/28), and sudden death (Oyo: 2/11; Kano: 0/28). Twelve of the farms (Oyo: 4/11; Kano: 8/28) reported no clinical signs. Additionally, farmers also indicated if they suspected disease on their farms. ND was suspected in a total of eight flocks (Oyo: 1, Kano: 7), IBD in three flocks (Oyo: 0, Kano: 3), and IB was not suspected in any of the flocks.

### Flock Vaccination Records

The vaccination history for each disease (ND, IBD, and IB) for each study flock was reported by the farmer, either via recall and/or from available records. All farmers reported administering ND vaccines. Some farmers did not provide details on IBDV or IBV vaccination records, and therefore it was assumed that these flocks were not vaccinated against these diseases. All farmers in Kano, and 4/16 (25%) farmers in Oyo, vaccinated their study flocks against IBDV (Table 2; Supplementary Table 3; Supplementary File 1). Conversely, more farmers in Oyo reported IBV vaccination [14/16 (87.5%)] than in Kano State [7/28 (25%)]. The proportion of flocks in Kano that received doses of IBDV vaccine was higher than in Oyo State (*p* < 0.001), while the proportion of flocks in Oyo that received doses of IBV vaccine was higher than in Kano (*p* < 0.001). There was also a significant difference in the number of NDV vaccine doses received between layer and broiler flocks (*p* < 0.001), but not for IBDV (*p* = 0.087) and IBV (*p* = 0.052) vaccines.

**Table 2:** Descriptive analysis of study flocks, evaluating associations between select flock vaccination variables and cases of NDV, IBDV, and IBV as determined by rRT-PCR.

**Variable**	**Response**	**Kano (** ***n*** **=** **28)**				**Oyo (** ***n*** **=** **16)**				**Total (** ***n*** **=** **44)**						
		**Freq**	**NDV cases (row %)**	**IBDV cases (row %)**	**IBV cases (row %)**	**Freq**	**NDV cases (row %)**	**IBDV cases (row %)**	**IBV cases (row %)**	**Freq**	**NDV cases (row %)**	**NDV pos/neg *p-*value*^a^***	**IBDV cases (row %)**	**IBDV pos/neg *p-*value*^a^***	**IBV cases (row %)**	**IBV pos/neg *p-*value*^a^***
NDV: Number of	Yes	28	5 (17.9)	–	–	16	2 (12.5)	–	–	44	7 (15.9)	<0.001	–	–	–	–
farms vaccinated	No	0	0	–	–	0	0	–	–	0	0		–		–	
Number of NDV	1	1	0	–	–	16	2 (12.5)	–	–	17	2 (11.8)	0.466	–	–	–	–
vaccine doses	2	7	2 (28.6)	–	–	0	0	–	–	7	2 (28.6)		–		–	
	3	13	1 (7.7)	–	–	0	0	–	–	13	1 (7.7)		–		–	
	4	7	2 (28.6)	–	–	0	0	–	–	7	2 (28.6)		–		–	
NDV: Vaccine strain	Komarov (mesogenic)	5	2 (40.0)	–	–	3	1 (33.3)	–	–	8	3 (37.5)	0.075	–	–	–	–
given^b^	Unspecified mesogenic	0	0	–	–	5	0	–	–	5	0		–		–	
	R2B (mesogenic)	0	0	–	–	3	1 (33.3)	–	–	3	1 (33.3)		–		–	
	LaSota (lentogenic)	23	5 (21.7)	–	–	3	0	–	–	26	1 (3.8)		–		–	
	B1 (lentogenic)	6	2 (33.3)	–	–	0	0	–	–	6	2 (33.3)		–		–	
	VH (lentogenic)	5	0	–	–	1	0	–	–	6	0		–		–	
	Not specified	0	0	–	–	1	0	–	–	1	0		–		–	
NDV: GMT antibody	<2,000	7	1 (14.3)	–	–	1	1 (100.0)	–	–	8	2 (25.0)	0.242	–	–	–	–
ELISA titer^c^	2,000– <8,000	11	3 (27.3)	–	–	6	1 (16.7)	–	–	17	4 (23.5)		–		–	
	≥8,000	10	1 (10.0)	–	–	9	0	–	–	19	1 (5.3)		–		–	
NDV: Protection	Protected	6	0	–	–	9	0	–	–	15	0	0.101	–	–	–	–
	Not protected	22	5 (22.7)	–	–	7	2 (28.6)	–	–	29	7 (24.1)		–		–	
IBDV: Number of	Yes	28	–	11 (39.3)	–	4	–	1 (25.0)		32	–	–	12 (37.5)	1.000	–	–
farms vaccinated	No	0	–	0	–	12	–	4 (33.3)		12	–		4 (33.3)		–	
Number of IBDV	0	0	–	0	–	12	–	4 (33.3)	–	12	–	–	4 (33.3)	0.829	–	
vaccine doses	1	0	–	0	–	4	–	1 (25.0)	–	4	–		1 (25.0)		–	
	2	28	–	11 (39.3)	–	0	–	0	–	28	–		11 (39.3)		–	
IBDV: Vaccine	Intermediate	14	–	6 (42.9)	–	0	–	0	–	14	–	–	6 (42.9)	0.966	–	
strain given^b^	Intermediate Plus	4	–	2 (50.0)	–	4	–	1 (25.0)	–	8	–		3 (37.5)		–	
	Virgo 7	7	–	3 (42.9)	–	0	–	0	–	7	–		3 (42.9)		–	
	Not specified	3	–	0	–	0	–	0	–	3	–		0		–	
IBDV: GMT antibody	<2,000	4	–	1 (25.0)	–	2	–	1 (50.0)	–	6	–	–	2 (33.3)	1.000	–	
ELISA titer^c^	≥2,000	24	–	10 (41.7)	–	14	–	4 (28.6)	–	38	–		14 (36.8)		–	
IBDV: Protection	Protected	21	–	7 (33.3)	–	14	–	4 (28.6)	–	35	–	–	11 (31.4)	0.340	–	
	Not protected	7	–	4 (57.1)	–	2	–	1 (50.0)	–	9	–		5 (55.6)		–	
Number of IBV	0	21	–	–	20 (95.2)	2	–	–	1 (50.0)	23	–	–	–		21 (91.3)	0.188
vaccine doses	1	7	–	–	5 (71.4)	14	–	–	10 (71.4)	21	–		–		15 (71.4)	
IBV: Vaccine	H120	4	–	–	2 (50.0)	4	–	–	2 (50.0)	8	–	–	–		4 (50.0)	0.184
strain given^b^	D274 clone	0	–	–	0	1	–	–	1 (100.0)	1	–		–		1 (100.0)	
	Not specified	3	–	–	3 (100.0)	10	–	–	8 (80.0)	13	–		–		11 (84.6)	
IBV: GMT antibody ELISA	<6,000	16	–	–	15 (93.8)	5	–	–	4 (75.0)	21	–	–	–		19 (90.5)	0.292
titer^c^	6,000– <15,000	8	–	–	6 (75.0)	5	–	–	3 (60.0)	13	–		–		9 (69.2)	
	≥15,000	4	–	–	4 (100.0)	6	–	–	4 (66.7)	10	–		–		8 (80.0)	
IBV: Protection	Protected	1	–	–	0	0	–	–	0	1	–	–	–		0	0.292
	Not protected	27	–	–	25 (92.6)	16	–	–	11 (68.8)	43	–		–		36 (83.7)	

a *p-value according to Pearson's Chi-squared test*.

b *Vaccine strain given was as reported/specified by the farmer. More than one vaccine strain may have been administered on one farm*.

c *Geometric mean (GMT) antibody titer is grouped by recommended minimum titers for the specified vaccines*.

A total of 34 different vaccines were reported to have been used on the study flocks in Kano and Oyo states. The strains covered by the vaccines and the number of farms administering these vaccines are detailed in Table 2 and Supplementary File 1. NDV LaSota, IBDV Intermediate, and IBV H120 strains were the most administered in Kano State, whereas in Oyo, NDV mesogenic, IBDV Intermediate Plus, and unspecified IBV vaccines were the most administered vaccines. The most administered vaccines to both layer and broiler flocks were NDV LaSota, IBDV Intermediate and unspecified IBV vaccines. Additionally, seven farmers (in Kano) reported the administration of multiple NDV strains to their birds. All IBDV vaccines were administered via drinking water, whereas NDV and IBV vaccines were administered through a variety of routes, including via drinking water, intra-ocularly, intra-muscularly, and per os, depending on the vaccine strain and manufacturer's instructions.

### Serology Results and Interpretation of Flock-Level Protection Against ND, IBD, and IB

As described above, protection of the study flocks against ND, IBD, and IB at the time of sampling was determined based on selected parameters. A flock-level summary of the interpretation of findings for each pathogen (NDV, IBDV, and IBV) based on the defined criteria is provided in Supplementary File 1.

#### NDV

**Figure 2:**
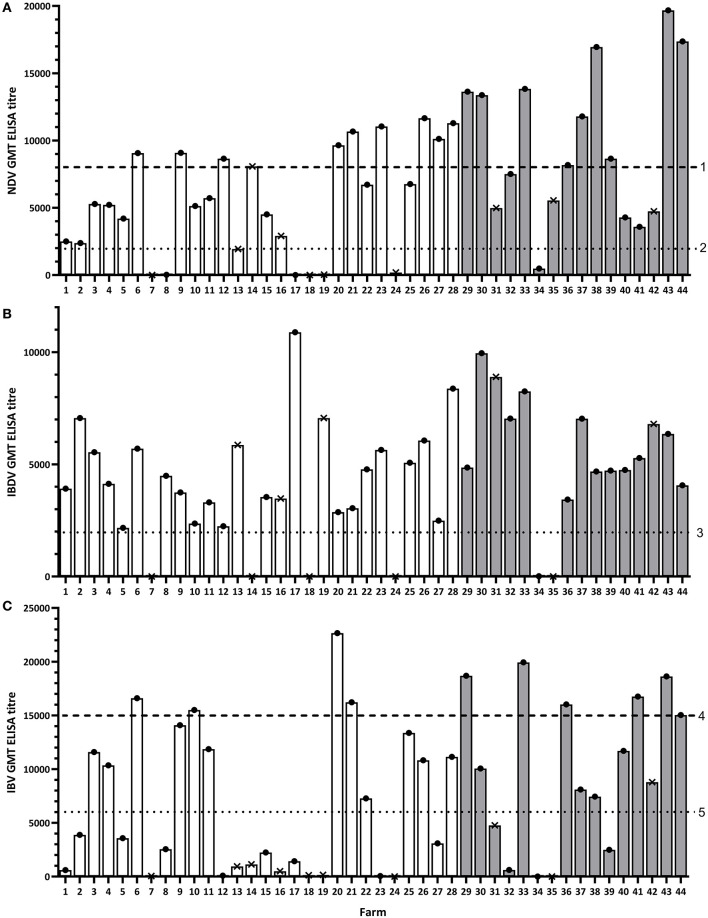
Geometric mean antibody titer (GMT) results for each study flock for NDV **(A)**, IBDV **(B)**, and IBV **(C)**. Dashed lines represent the recommended minimum titer that is considered protective after administration of the specified vaccine in the study region: 1, ND killed virus vaccine; 2, ND live virus vaccine; 3, IBD live/killed virus vaccine; 4, IB killed virus vaccine; 5, IB live virus vaccine. White bars represent flocks in Kano, gray bars represent flocks in Oyo. Dots and crosses at the top of bars represent layer and broiler flocks, respectively.

A total of 15/16 (93.8%) flocks in Oyo and 21/28 (75%) in Kano had an NDV GMT antibody titer above the recommended minimum titer (2,000) for the ND live virus vaccine (Figure 2; Table 2; Supplementary File 1). More layer flocks (31/33, 93.9%) than broiler flocks (3/9, 33.3%) had an NDV GMT antibody titer above 2,000 (*p* < 0.001). There was only one flock where no birds tested positive by the NDV antibody ELISA (titer = 0).

Additionally, a total of 9/16 (56.3%) flocks in Oyo and 10/28 (35.7%) in Kano had an NDV GMT antibody titer above 8,000 for the ND killed virus vaccine (Figure 2; Table 2; Supplementary File 1). Again, more layer flocks (18/33, 54.6%) than broiler flocks (1/9, 11.1%) had an NDV GMT antibody titer above 8,000 (*p* = 0.052).

The proportion of flocks considered as protected against ND was significantly different between Oyo (9/16, 56.3%) and Kano states (6/28, 21.4%) (*p* = 0.044), but not between layer (13/33, 39.4%) and broiler bird types (2/9, 22.2%) (*p* = 0.575) (Table 3; Supplementary File 1). In Oyo state, 8/12 (66.7%) layer flocks and 1/2 (50.0%) broiler flocks were considered as protected, and in Kano state, 5/21 (23.8%) layer flocks, and 1/7 (14.3%) broiler flocks were considered protected.

**Table 3:** Descriptive analysis of study flocks, evaluating associations between select flock variables and flock-level protection against NDV, IBDV, and IBV.

**Variable**	**Response**	***N***	**NDV**			**IBDV**			**IBV**		
			**Protected (%)**	**Not protected (%)**	***p*-value^a^**	**Protected (%)**	**Not protected (%)**	***p*-value^a^**	**Protected (%)**	**Not protected (%)**	***p*-value^a^**
State	Kano	28	6 (21.4)	22 (78.6)	0.044	21 (75.0)	7 (25.0)	0.548	1 (3.6)	27 (96.4)	1.000
	Oyo	16	9 (56.3)	7 (43.8)		14 (87.5)	2 (12.5)		0	16 (100.0)	
Bird type	Broiler	9	2 (22.2)	7 (77.8)	0.366	2 (22.2)	7 (77.8)	<0.001	0	9 (100.0)	0.843
	Layer	33	13 (39.4)	20 (60.6)		31 (93.9)	2 (6.1)		1 (3.0)	32 (97.0)	
	Broiler-breeder	2	0	2 (100.0)		2 (100.0)	0		0	2 (100.0)	
Flock size	<200	1	0	1 (100.0)	0.377	0	1 (100.0)	0.096	0	1 (100.0)	0.627
	200– <500	7	1 (14.3)	6 (85.7)		5 (71.4)	2 (28.6)		0	7 (100.0)	
	500– <2,000	10	2 (20.0)	8 (80.0)		8 (80.0)	2 (20.0)		1 (10.0)	9 (90.0)	
	2,000– <5,000	12	6 (50.0)	6 (50.0)		11 (91.7)	1 (8.3)		0	12 (100.0)	
	5,000– <15,000	13	6 (46.2)	7 (53.8)		11 (84.6)	2 (15.4)		0	13 (100.0)	
	≥15,000	1	0	1 (100.0)		0	1 (100.0)		0	1 (100.0)	
Mortality/week	<5	23	7 (30.4)	16 (69.6)	0.327	19 (82.6)	4 (17.4)	0.319	1 (4.3)	22 (95.7)	0.968
	5–9	8	4 (50.0)	4 (50.0)		7 (87.5)	1 (12.5)		0	8 (100.0)	
	10–19	3	0	3 (100.0)		2 (66.7)	1 (33.3)		0	3 (100.0)	
	20–49	7	4 (57.1)	3 (42.9)		6 (85.7)	1 (14.3)		0	7 (100.0)	
	50–99	1	0	1 (100.0)		0	1 (100.0)		0	1 (100.0)	
	≥100	2	0	2 (100.0)		1 (50.0)	1 (50.0)		0	2 (100.0)	

a *p-value according to Pearson's Chi-squared test*.

#### IBDV

A total of 14/16 (87.5%) flocks in Oyo and 24/28 (85.7%) in Kano had an IBDV GMT antibody titer above the recommended minimum titer for the IBD live virus vaccine (titer = 2,000) (Figure 2; Table 2; Supplementary File 1). More layer flocks (33/33, 100%) than broiler flocks (3/9, 33.3%) had an IBDV GMT antibody titer above 2,000 (*p* < 0.001). There were two broiler flocks in Kano where none of the birds sampled tested positive by the IBDV antibody ELISA (titer = 0). For all flocks with no reported record of IBDV vaccination (all in Oyo), 12/12 (100%) had a GMT antibody titer above the recommended minimum titer (2,000).

Of the 16 flocks in Oyo, 14 (87.5%) were considered as protected against IBD (Table 3; Supplementary File 1). These 14 flocks (12 layer flocks and 2 broiler-breeder flocks) housed birds over 16 weeks of age and were therefore not considered susceptible to IBD due to the expected regression of the bursa by this age. The two broiler flocks in Oyo that were not considered as protected housed birds aged 2 and 3 weeks (Supplementary File 1).

In Kano, a total of 21/28 (75%) flocks were considered as protected against IBD (19 layer flocks, two broiler flocks) (Table 3, Supplementary File 1). For those flocks where the ages of birds were known, 14/28 (50%) flocks were layer birds over 16 weeks of age where birds were no longer susceptible to IBD. For the remaining 12 flocks where birds were <16 weeks of age (five layer flocks, seven broiler flocks), seven flocks were not considered as protected against IBD (two layer flocks, five broiler flocks). Two layer-flocks did not specify the age of their birds, but it was assumed they were older than 16 weeks based on the vaccination record provided by the farmer.

The number of flocks considered as protected against IBD was significantly different between the bird types (*p* < 0.001), and across the flock size categories for layer birds (*p* = 0.002) but not for broilers (*p* = 0.733), or between the two states (*p* = 0.548) (Table 3).

#### IBV

A total of 11/16 (68.8%) flocks in Oyo and 12/28 (42.9%) in Kano had an IBV GMT antibody titer above 6,000 for the IB classic strain live virus vaccine (Figure 2; Table 2; Supplementary File 1). More layer flocks (22/33, 66.7%) than broiler flocks (0/9, 0.0%) had an IBV GMT antibody titer above 6,000 (*p* = 0.002).

Additionally, 6/16 (37.5%) flocks in Oyo and 4/28 (14.3%) in Kano had an IBV GMT antibody titer above 15,000 for the classic strain killed virus vaccine and the variant strain vaccine (Figure 2; Table 2; Supplementary File 1). More layer flocks (10/33, 30.3%) than broiler flocks (0/9, 0.0%) had an IBV GMT antibody titer above 15,000 (*p* = 0.147).

None of the flocks in Oyo, and only one layer flock in Kano (3.6%), were considered as protected against IB (Table 3; Supplementary File 1).

### rRT-PCR Results

#### NDV

NDV RNA was detected on 7/44 (15.9%) farms (Tables 1, 4; Supplementary Figure 1; Supplementary File 1). NDV RNA was detected on more farms in Kano (5/28, 17.9%) than in Oyo (2/16, 12.5%), and in a higher proportion of broiler flocks (3/9, 33.3%) than layer flocks (4/33, 12.1%), although these differences were not significant. In Oyo, NDV RNA was detected in 1/12 (8.3%) layer flocks and 1/2 (50.0%) broiler flocks. In Kano, NDV RNA was detected in 3/21 (14.3%) layer flocks and 2/7 (28.6%) broiler flocks. There was a significant difference between the age categories of layer flocks where NDV RNA was detected (*p* = 0.001) (Table 1).

**Table 4:** rRT-PCR results by tissue type tested.

**Virus**	**Total (*n* = 44)**	**Tissue type tested**			
		**Bursal tissue^a^**	**Cloacal swab and proventriculus^a^**	**Tracheal swab and oropharyngeal swab^a^**	**Caecal tonsil^a^**
NDV positive	7 (15.9%)	–	5/7 (71.4%)	6/7 (85.7%)	–
IBDV positive	16 (36.4%)	16/16 (100.0%)	–	–	–
IBDV_nv	3/16 (18.8%)	3/16 (18.8%)	–	–	–
IBDV_vv	3/16 (18.8%)	3/16 (18.8%)	–	–	–
IBV positive	36 (81.8%)	–	–	23/36 (63.9%)	35/36 (97.2%)
IBV 4/91	1/36 (2.3%)	–	–	1/23 (4.3%)	0
IBV Massachusetts	2/36 (5.6%)	–	–	1/23 (4.3%)	1/35 (2.9%)
IBV Variant 02	11/36 (30.6%)	–	–	6/23 (26.1%)	11/35 (31.4%)
IBV D1466	0	–	–	0	0
IBV D274	0	–	–	0	0
IBV Italy 02	0	–	–	0	0
IBV Arkansas	0	–	–	0	0
IBV IB80	0	–	–	0	0
IBV Q1	2/36 (5.6%)	–	–	0	2/35 (5.7%)
IBV QX	0	–	–	0	0
IBV: typing not possible	21/36 (58.3%)	–	–	15/23 (65.2%)	21/35 (60.0%)

–*: Not done—sample type was not tested for this virus*.

a *Each tissue type was collected from two birds per farm and pooled as one sample. Samples consisting of two tissue types are pooled from the same two birds*.

Of the seven NDV positive samples, NDV RNA was identified in both pooled sample types on four farms, in the cloaca/proventriculus only on one farm in Oyo, and in tracheal/oropharyngeal swabs only on two farms in Kano (Table 4; Supplementary Figure 1).

NDV RNA was not detected in any of the flocks considered as protected against ND in Oyo or Kano.

#### IBDV

IBDV RNA was detected by the screening rRT-PCR assay on 16/44 (36.4%) farms; on more farms in Kano State (11/28, 39.3%) than in Oyo (5/16, 31.3%) (Tables 1, 4; Supplementary Figure 1; Supplementary File 1), and in a higher proportion of broiler flocks (5/9, 55.6%) than layer flocks (10/33, 30.3%), although these differences were not significant. In Oyo, IBDV RNA was detected in 3/12 (25.0%) layer flocks, 1/2 (50%) broiler flocks, and 1/2 (50.0%) broiler-breeder flocks. In Kano, IBDV RNA was detected in 7/21 (33.3%) layer flocks, and 4/7 (57.1%) broiler flocks. Additionally, IBDV RNA was detected in more younger layer flocks than older flocks (*p* = 0.004), and a significant difference was observed in the detection of IBDV RNA between levels of bird mortality (*p* = 0.030) (Table 1).

Of the IBDV positive samples (*n* = 16), the very virulent (including the intermediate plus) strain (vvIBDV) was identified in three bursal tissue samples [Oyo = 1 (broiler flock), Kano = 2 (1 broiler flock, 1 layer flock)]. The non-virulent (including the intermediate) strain (nvIBDV) was identified in three bursal tissue samples (one broiler flock, two layer flocks), all from farms in Oyo state (Table 4; Supplementary Figure 1). A total of 10/16 IBDV positive samples could not be detected by either the vvIBDV or nvIBDV pathotyping assays.

Of the flocks that were considered as protected against IBD, IBDV RNA was detected in 4/13 flocks [30.8%, (one broiler-breeder flock, three layer flocks)] in Oyo and 7/21 [33.3%, (two broiler flocks, five layer flocks)] broiler flock in Kano (Table 1; Supplementary File 1).

#### IBV

IBV RNA was detected on 36/44 (81.8%) farms; on more farms in Kano (25/28, 89.3%) than in Oyo (11/16, 68.8%) (Tables 1, 4; Supplementary Figure 1; Supplementary File 1), and in a higher proportion of broiler flocks (8/9, 88.9%) than layer flocks (26/33, 78.8%), although these differences were not significant. In Oyo, IBV RNA was detected in 8/12 (66.7%) layer flocks, 1/2 (50.0%) broiler flocks, and 2/2 (100.0%) broiler-breeder flocks. In Kano, IBV RNA was detected in 18/21 (85.7%) layer flocks, and 7/7 (100.0%) broiler flocks.

Of the 36 IBV positive samples, IBV RNA was identified in both sample types on 22 farms (Table 4; Supplementary Figure 1), only in the pooled tracheal/oropharyngeal swab on one farm, and in only the cecal tonsil on 13 farms.

Four strains were detected in the birds sampled (Table 4; Supplementary Figure 1). These included Variant02 which was detected on 11 farms (three broiler flocks, eight layer flocks) in Kano; Massachusetts, which was detected in one broiler flock in Kano and one broiler farm in Oyo; 4/91, detected in one layer flock in Oyo; and Q1, detected in one broiler-breeder layer flock in Oyo. One sample collected in a broiler flock in Kano was positive for both Variant02 and Massachusetts strains. The strains of IBV could not be typed in samples from 7/11 (63.6%) farms where IBV was detected in Oyo, and 14/25 (56.0%) farms in Kano.

IBV RNA was not detected in any samples from the one layer flock considered to be protected against IB.

**Figure 3:**
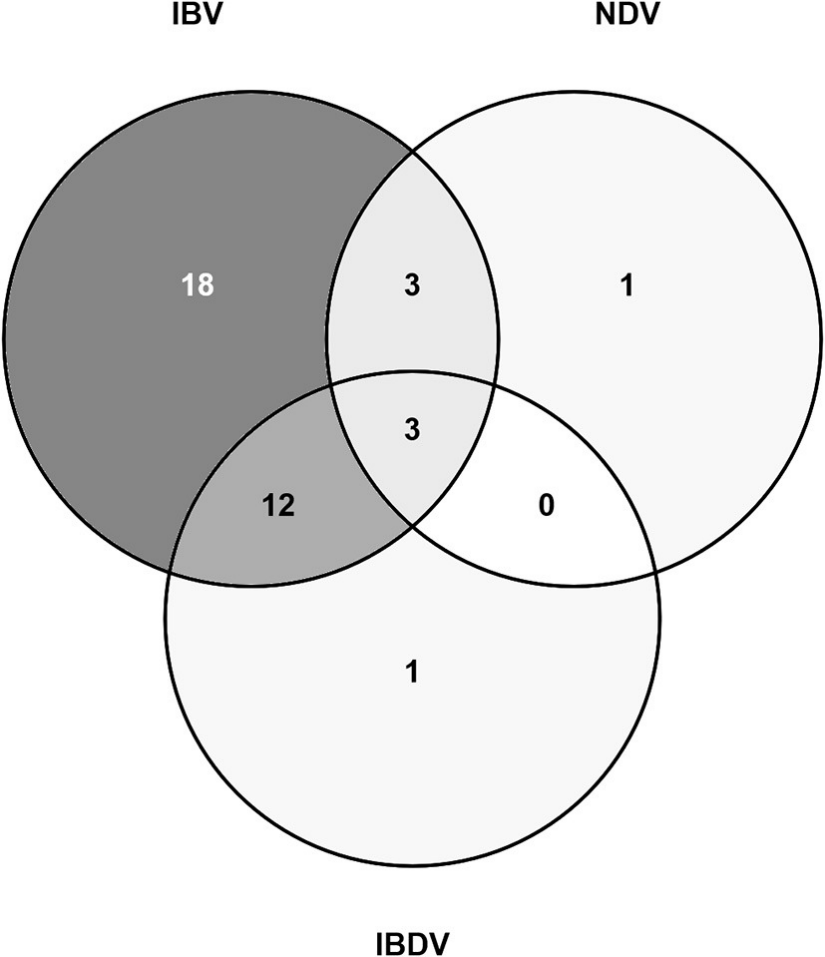
Venn diagram showing the number of study flocks where a sample tested positive for NDV, IBDV, and/or IBV RNA by rRT-PCR.

Both IBV and NDV RNA were detected on the same six farms [Oyo:1 (layer flock), Kano:5 (two broiler flocks, three layer flocks)], and 5/6 of these were detected in the same pooled samples (tracheal and oropharyngeal swab) (Figure 3). Both IBV and IBDV RNA were detected on the same 15 farms [Oyo: 4 (one broiler flock, one broiler-breeder flock, two layer flocks), Kano:11 (four broiler flocks, seven layer flocks)]. Both NDV and IBDV RNA were detected on the same three farms [Kano: 3 (one broiler flock, two layer flocks)]. There were three farms (Farms 2, 15, and 16) where all three viruses were detected by rRT-PCR.

### Sequence Analysis

#### NDV

Sequences were obtained from the pooled oropharyngeal and tracheal swab samples collected from two farms (Farm 2, sample ref: A1920457.030 and 7, sample ref: A1920457.035) in Kano and one farm (Farm 34, sample ref: A1917188.022) in Oyo, that were positive by the NDV rRT-PCR. Based on the sequences of the fusion (*F*) protein gene, the sequenced strains were assigned to the lentogenic PMV-1 strains. The NDV sequences were not of sufficient quality to perform phylogenetic analyses. (Sequences are soon to be submitted to GenBank, NCBI to obtain accession numbers).

#### IBDV

**Figure 4:**
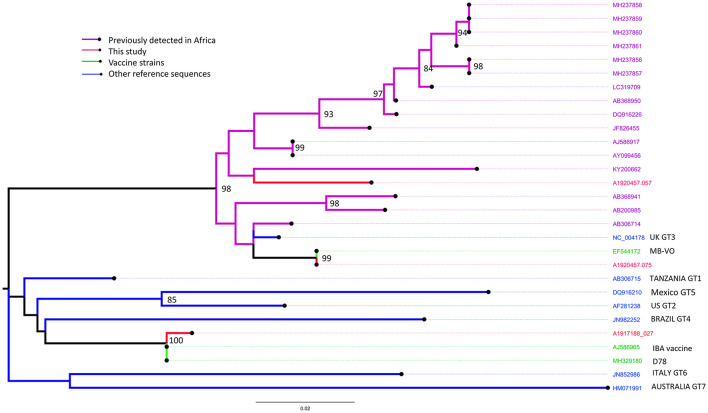
Maximum likelihood phylogenetic tree of a 394 nucleotide region of 29 sequences showing the relationships between the IBDV isolates obtained from Farms 1 (sample ref: A1920457.057), 19 (A1920457.075) and 35 (A1917188.027) for the *VP2* gene implemented in MEGA-X. Study sequences are shown in red. Bootstrap values (percentage of 500) over 85 are shown. GT: genotype (GT1, classical pathotype; GT2, antigenic variant; GT3, vvIBDV; GT4, dIBDV; GT5, variant/classical recombinant; GT6, Italy; GT7, Australian).

*VP1* and *VP2* sequences were obtained from the bursa samples collected from two farms (Farm 1 and 19) in Kano (identified by RT-PCR as from the vvIBDV strain) and only the *VP2* sequence from one farm (Farm 35) in Oyo (identified as from the nvIBDV strain), that were positive by the IBDV rRT-PCR. In comparison with known vaccine and field strains, the *VP2* sequences obtained from isolated RNA from Farm 1 (sample ref: A1920457.057) clusters with the group of genotype 3 virulent IBDV strains previously reported from Africa (Figure 4).

In comparison with known vaccine and field strains the sequence obtained from Farm 19 (sample ref: A1920457.075) was identical to the MB vaccine strain, and the sequence obtained from Farm 35 (sample ref: A1917188.027) was most closely related to the classically attenuated vaccine strain D78 (Figure 4).

#### IBV

Sequences were obtained from two pooled oropharyngeal and tracheal swab samples (Farm 16: Kano, Farm 35: Oyo) and three cecal tonsil samples (Farms 7, 11, and 12: Kano) that were positive by the IBV rRT-PCR.

**Figure 5:**
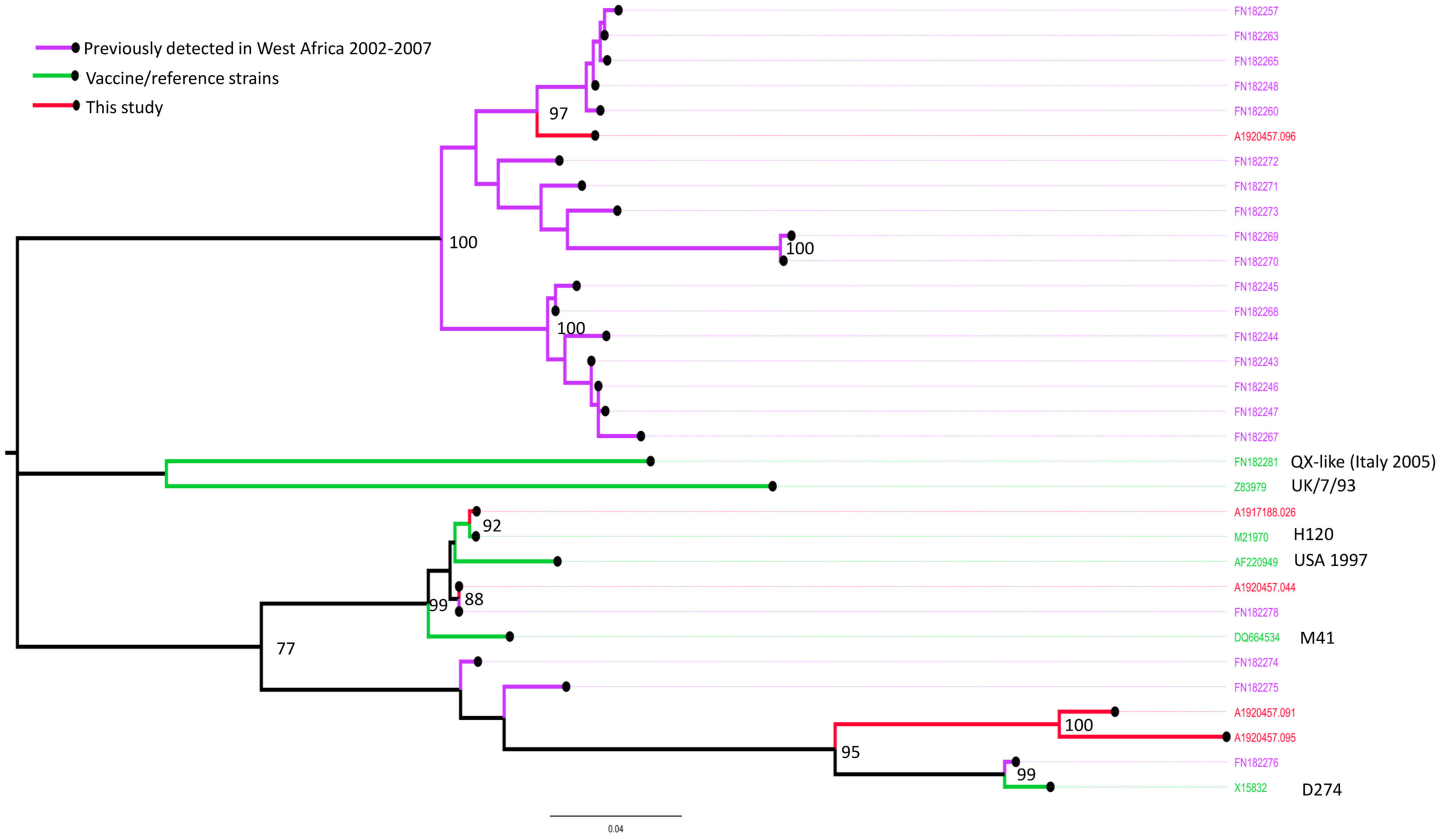
Maximum likelihood phylogenetic tree of a 470 nucleotide region of 32 sequences implemented in MEGA-X, showing the relationships between the IBV isolates obtained from Farms 7 (sample ref: A1920457.091), 11 (A1920457.095), 12 (A1920457.096), 16 (A1920457.044), and 35 (A1917188.026), based on the amplified partial nucleotide sequence coding for the S1-spike protein of Infectious Bronchitis Virus (IBV) and selected reference sequences. Study sequences are shown in red. Bootstrap values (percentage of 500) over 85 are shown.

Based on phylogenetic analysis of the *S1*-spike protein of IBV, the sequences obtained from Farm 16 (sample ref: A1920457.044) and Farm 35 (sample ref: A1917188.026) clustered with the Massachusetts strains M41 and H120, commonly used in live IBV vaccines (up to 100.0% nucleotide identity over the region studied) (Figure 5). Samples from farms 16 and 35 were also positive on the Massachusetts IBV strain-specific rRT-PCR assay, and Farm 16 was also positive on the Variant 02 assay.

The sequences obtained from Farm 7 (sample ref: A1920457.091) and Farm 11 (sample ref: A1920457.095) were similar, and most closely related to the D274-like strains, including one isolate previously detected in Nigeria in 2006. Samples from farms 7 and 11 were positive on the Variant 02 strain-specific rRT-PCR assay.

The sequence obtained from Farm 12 (sample ref: A1920457.096) was most closely related to a clade of sequences detected in commercial and back yard poultry in Nigeria and Niger in 2006 and 2007 (19). The sample from farm 12 was not positive on any of the strain-specific rRT-PCR assays.

## Discussion

Despite the use of vaccination programs, ND, IBD, and IB continue to severely limit the poultry industry in Nigeria. This study aimed to assess the level of protection against ND, IBD, and IB currently afforded by routine vaccination practices on commercial poultry farms. The intent was to evaluate the serology results including the GMT antibody titers, not in isolation, but with respect to vaccination and select relevant parameters as described previously in the methods section, to determine if a given flock had adequate protection against each disease at the time of sampling. Additionally, this study aimed to characterize circulating strains of NDV, IBDV, and IBV, so that the results may inform effective vaccine selection.

### NDV

All farms visited in Kano and Oyo states reported administering NDV vaccines, and as expected, the number of doses of vaccine administered varied by bird type (broilers vs. layers). The differences in the number of doses of vaccine administered by bird type may be due to differences in the age of the birds at the time of the study visit, differences in timing of routine vaccination on each farm, and/or revaccination conducted following suspicion of NDV outbreaks on some farms. However, it is unknown why there was a significant difference between the number of NDV vaccine doses between the two states. The LaSota NDV vaccine strain was the most commonly administered, on 86.4% of farms in the study areas. The reasons for this finding are not clear, it may be that this vaccine was the most easily obtained, or the most cost-effective, hence the high uptake of this vaccine strain. Other NDV strains including Hitchner B1 and Komarov were also reported in the current study. Vaccination schedules that involve the administration of Hitchner B1, LaSota, and Komarov strains have been reported in Nigeria (27, 28), and combinations of these strains as live and inactivated oil-emulsion vaccines have been shown to induce high NDV antibody titers indicative of protection (27, 29).

However, despite the high uptake of NDV vaccination on the study farms, only 15/44 (34.1%) flocks were considered as protected against ND, with the remaining flocks considered as not protected and therefore at risk of exposure to NDV field challenge. Interestingly, the number of flocks considered as protected against ND was significantly higher in Oyo (56.3%) than in Kano state (21.4%). There was no significant difference in the protection status by bird type. Newcastle disease viral RNA was not detected in any of the 15 flocks that were considered protected but was detected in 7/29 (24.1%) flocks considered not protected; 5/28 (17.9%) in Kano and 2/16 (12.5%) in Oyo, and in more broiler flocks than layer flocks, although these differences were not significant. Previous studies conducted on individual chickens (rather than by flock) in other regions of Nigeria have reported isolation of NDV from 3.2% of healthy, unvaccinated commercial poultry in Nsukka, Enugu state in the South (30) and from 30.8% of domestic chickens in Yobe state in the North (31). In the current study, a higher proportion of farms in the North were reported to have circulating NDV compared to the South of Nigeria, and this was supported by the serology data. The reasons for these regional differences are not clear but may be due to potential differences in management; farms in the south are larger and therefore expected to have better developed and organized vaccination programs.

Following detection of NDV RNA by real-time RT-PCR, sequences identified as the lentogenic PMV-1 strain were obtained from samples collected from two flocks in Kano state with broiler and layer flocks and one broiler flock in Oyo. Lentogenic strains of NDV have been identified and characterized from samples collected in Oyo and Kano states previously (15, 32). None of these three affected flocks (Farms 2, 7, and 34) were considered as protected against NDV despite receiving prior NDV vaccination, leaving the birds vulnerable to field challenge. At least two doses of the Izovac live vaccine, containing the LaSota strain (a lentogenic strain), had reportedly been administered to both flocks in Kano (Farms 2 and 7), and one dose of the Hester NDV live vaccine, containing the R2B strain (a mesogenic strain) was reported to have been administered to the flock in Oyo (Farm 34). It is likely that the strain detected on Farm 34 was the result of a field challenge, due to the difference in strain types between what was detected (lentogenic) and that vaccinated against (mesogenic), although the farmer did not report any clinical signs or mortalities. This highlights the importance of understanding circulating strains in a region, so that vaccination programs can be suitably informed and utilized. On Farms 2 and 7 at the time of sampling, farmers reported respiratory distress, and mortalities of three and two birds per week, respectively. On Farm 7, and as assumed on Farm 2 (due to incomplete reporting), the last dose of live NDV vaccine had been administered <10 days previously, and consequently it is possible that birds may have been clinically affected by, and/or were excreting vaccine virus, hence the lentogenic strain observed via sequencing. Indeed, previous reports have indicated that viral replication and excretion may occur due to the vaccine virus itself, consequently resulting in subclinical infection (30, 33). Additionally, in the current study, co-infections of NDV with IBV were observed in 6 flocks, as has been previously reported (34, 35). Also, co-infections of NDV and IBDV were also observed in three flocks, which may be the first observation of this in Nigeria. Therefore, it is possible that any clinical signs/death observed may have instead been due to IB or IBD.

### IBDV

In Kano and Oyo states, 79.5% (35/44) of the flocks were considered as protected against IBD. Although all farms in Kano reported vaccinating their birds with IBDV vaccine, only 75% (21/28) of these flocks were considered as protected; 19/21 were layer flocks and 2/21 were broiler flocks. Many of the flocks (57.1%) were older than 16 weeks at the time of sampling, and were therefore considered protected, as they were no longer susceptible due to regression of the bursa by this age (36). Seven of the 28 flocks in Kano were considered not protected against IBD; five were broiler flocks aged between 4 and 7 weeks, while two were layer flocks aged 6 and 7 weeks. In Oyo state, only 25% (4/16) of farms reported administering IBDV vaccine, however, 14/16 flocks (2 broiler-breeder flocks and 12 layer flocks) were considered protected due to the age of the birds, and all of these had GMT antibody titers above the recommended minimum. The 2/16 flocks considered not protected were broiler flocks aged 2 and 3 weeks.

All nine flocks considered not protected against IBD across both states were therefore considered at risk of exposure to IBDV field challenge. IBDV RNA was detected on five of these farms [Farm 35 in Oyo (nvIBDV) and Farms 1 (vvIBDV), 8, 16, and 24 in Kano], suggesting there was circulating IBD virus on the study farms at the time of the sampling. This was supported by sequencing results, where sequences from Farm 1 were most closely related to a clade of vvIBDV previously reported in Africa (37). Additionally, the *VP2* sequence from this farm was closely related to reported strains belonging to a unique cluster of northwest Nigerian field IBDV strains isolated from Kaduna and Katsina states, both of which neighbor Kano state region (38–40). The detection of the *VP2* sequence on Farm 1, in a similar geographical location to previous reports suggests local circulation of this strain in this region. This farm had administered two doses of live IBDV vaccine, but not all birds had sufficient antibody titers, and the farmer reported high levels of bird mortality in the flock and suspected an IBD outbreak. The vaccine reported to have been used on this farm was the Cevac live vaccine containing the 2,512 strain and was not closely related to these northern Nigerian strains, and therefore antigenic difference may be the reason for vaccine failure in this flock. Therefore, the choice of vaccine should be carefully considered by veterinarians and farmers based on the known circulating strains in a region.

Both pathotypes of IBDV (nvIBDV and vvIBDV) were identified in Oyo state, whereas only the vv strain was identified in Kano state. This may suggest differences in viral circulation between the two regions; however, both strains have been reported in both Northern and Southern Nigeria (40). For example, the vv strain has previously been identified in Kaduna and Plateau states which are the Southern neighbors of Kano state (41, 42), and in Oyo state in Southern Nigeria (43). Unfortunately, some of the samples positive by the IBDV screening rRT-PCR assay could not be typed, and therefore it is difficult to rule out that some of these samples from Oyo were the vv strain.

Of the 35 flocks considered as protected against IBD, IBDV RNA was detected on four farms in Oyo and seven farms in Kano. Farm 19 in Kano State was the only farm considered as protected on which IBDV RNA (IBDV vv strain) was detected and a sequence could be obtained. This flock had birds aged 5 weeks and GMT IBDV antibody titers above the recommended minimum of 2000, with 18/18 (100%) birds above the minimum titer. These serology results indicate that previous vaccination on this farm was successful in stimulating a good antibody response. The IBDV *VP2* sequence obtained from RNA isolated from this farm (Farm 19) was 100% identical to the MB vaccine strain. This farm reported vaccination with two doses of the live Biovac (Fatro, Italy) vaccine containing the Virgo7 intermediate strain. It is possible that the farmer or supplier may have mistaken the vaccine strain given to the birds or there was a recall error, and the viral RNA detected may be vaccine derived, especially considering that the last vaccination was administered <2 weeks prior to sampling. Owoade et al. (43) reported that the MB vaccine was closely related to many IBDV strains isolated from Nigeria, and there have been many other reports of similar sequences of field isolates and IBDV vaccine strains (38, 44, 45). Inaccurate reporting may also have been the case on Farm 35, where the *VP2* sequence obtained was closely related to those of several vaccine strains. Alternatively, it is possible that these vaccine strains may be circulating in these flocks having potentially reverted to virulence. Adamu et al. suggested that IBDV strains may mutate, but maintain their identity on the *VP2* region (38). Additionally, challenges with storage or administration of the vaccine administered may have resulted in reduced vaccine effectiveness on this farm, allowing for the circulation of this strain. These findings highlight the need to conduct studies that investigate causes of vaccine failure on farms.

Interestingly, five of the flocks (one broiler-breeder and four layer flocks) considered as protected where IBDV RNA was detected were aged 18, 23, 26, 33, and 42 weeks old. This is surprising considering that birds aged 14–16 weeks or more are thought to be no longer susceptible to IBD due to regression of the bursa by this age (36). The detection of IBDV RNA in birds aged 16 weeks or more has been documented previously. In 1981 Okoye and Uzoukwu (46) observed IBDV in chickens aged 20 weeks old. In the current study, IBDV RNA was detected in one flock aged 42 weeks, which may be the oldest flock from which IBDV RNA has been reported to be detected. Sequences could not be obtained for the IBDV isolates from these five farms, however, it is possible that the viral RNA detected may have been present as a result of recent vaccination against IBDV, as was likely the case on the farm in Kano (Farm 19). Although IBDV vaccination records were not provided for four of these flocks (and therefore they were assumed not to be vaccinated), the GMT IBDV antibody titer was above the recommended minimum of 2000, and 18/18 birds (100%) for flocks aged 18, 23, 26, and 33 weeks, and 17/18 (94%) birds for the flock aged 42 weeks were above the minimum titer, suggesting that these flocks were actually vaccinated against IBDV.

Additionally, contamination of samples from the environment cannot be ruled out as a reason for detection of IBDV RNA in these flocks considered as protected, for example from viral RNA present in the air, dust, fecal matter, or bed litter (47, 48), especially on farms with inadequate biosecurity measures in place to stop the introduction of virus from external sources such as free-roaming village chickens.

### IBV

Less than 50% of farms reported IBV vaccination, and most of the flocks were not considered as protected against IB except for one (2.3%) in Kano. It is possible that farmers have little awareness of IB, which may also explain the low uptake of vaccination in the study areas. Interestingly, more farms in Oyo state vaccinated their birds against IBV than in Kano, in contrast to the IBDV vaccination uptake. The reasons for this difference are not clear, but it may be linked to the more advanced poultry industry in Oyo, which houses up to 70% of Nigeria's poultry, or to differences in vaccine market distribution, availability of diagnostic laboratories to diagnose IBV, veterinary provider awareness of IBV, and availability of extension services, with Oyo at an advantage compared to Kano.

One layer flock (Farm 3) in Kano was considered as protected against IB and IBV RNA was not detected in birds sampled on this farm. The GMT antibody titer and the titers of all the birds sampled in this flock were above the recommended minimum of 6,000 for IBV classic live strain. However, only 2/18 birds had an antibody titer above the recommended minimum of 15,000 for killed IBV vaccine. Although IBV vaccination records were not provided for this farm, the observed antibody titers suggest that this flock may have received at least one dose of IBV vaccine that was not reported by the farmer. It is possible that this may be due to misinformation or unawareness of multivalent vaccines, and therefore IBV may have been unknowingly present in a vaccine, that the farmer may have mistaken for a monovalent vaccine. Alternatively, it is possible that previous IBV field challenges had occurred resulting in birds producing high levels of IBV antibody. Indeed, some of the flocks that were not considered as protected had IBV GMT antibody titers above the recommended minimum titers of 6,000 and 15,000 after vaccination, despite no reported record of IBV vaccination.

IBV RNA was detected on 36/44 (81.8%) farms; detected on more farms in Kano (25/28, 89.3%) than in Oyo (11/16, 68.8%), and in a higher proportion of broiler flocks (8/9, 88.9%) than layer flocks (26/33, 78.8%) although these differences were not significant. Studies investigating viral prevalence of IB in Nigeria are limited, however, evidence of IBV has been detected in many states of Nigeria including Oyo, Ogun, Lagos, Kano, Kaduna, Sokoto, and Yobe states (19, 31), suggesting a wide distribution, and the requirement for further epidemiological investigation including the circulating strains.

Of interest in this study, there were several cases where IBV and NDV, IBV, and IBDV, or NDV and IBDV were detected on the same farms, as has been reported previously (35, 49). Additionally, in three flocks, co-infection of all three viruses was observed. Unfortunately, due to the pooling of samples, it is unknown whether these were all present in the same bird; however, presence of the three viruses in the same flock indicates the importance of an effective vaccination schedule, alongside other control measures such as farm biosecurity.

When IBV positive samples were typed using 10 variant-specific rRT-PCR assays, the Massachusetts strain was detected in both Kano and Oyo states. Interestingly however, Variant02 was detected in 11/25 (44.0%) IBV positive samples in Kano, but not in any of the 11 positive samples in Oyo, whereas 4/91 and Q1 variants were only detected in Oyo state, highlighting a potential regional difference in viral circulation. Unfortunately, sequences could not be obtained for many of the samples due to a low level of RNA, particularly those from Oyo typed as 4/91 or Q1, which would have been useful to provide more information on the source of these viral variants.

One pooled sample from a broiler flock (Farm 16) in Kano tested positive for both Variant02 and Massachusetts by variant-specific rRT-PCR. This isolate was identified through sequence analysis as being part of the Massachusetts variant, and clustered with vaccine strains M41 and H120. However, this farm had not reported vaccination against IBV and none of the birds on this farm had an IBV antibody titer over the recommended minimum for classic or variant strain vaccines, and therefore a natural infection in the flock at the time of sampling may have been likely in this flock. Indeed, the Massachusetts IBV variants have been reported to cause sporadic IB outbreaks in the commercial poultry industry in many African countries and may circulate sub-clinically (20, 21). Alternatively, it is possible that farmer reporting regarding vaccination was inaccurate, and that the viral RNA detected may have been vaccine derived. This may also have been the case on Farm 35 (in Oyo), where sequence analysis of this isolate was also typed as the Massachusetts variant and clustered with vaccine strains M41 and H120. Indeed, this farm had reported administering the Izovac IB H120 vaccine <2 weeks prior to the time of sampling. Evidence of the continuing evolution of IBV has been reported resulting in outbreaks in vaccinated poultry, including reversion of vaccine strains to virulence which may have occurred on these farms, and the recombination of several strains (50, 51).

On two other farms in Kano (Farms 7 and 11), IBV isolates detected were closely related to vaccine strain D274. This flock was not considered protected, and none of the birds had an IBV antibody titer over the recommended minimum for classic or variant strain vaccines. Persistence of the latter isolate has been reported in flocks vaccinated with H120 (52–54), and although this farm had no reported record of IBV vaccination, at least eight other farms in this study reported using this vaccine, and therefore it is possible that there was circulation of this vaccine strain, or RNA detected was derived from unknowingly administered vaccine.

On Farm 11, the flock had a GMT IBV antibody titer of >13,000, above the recommended minimum for the classic strains live vaccine, but below that for the IBV variant strain vaccines (15,000), and therefore was not considered protected. The sequence obtained from Kano (Farm 12) clustered as part of a new QX-like “IBADAN” genotype, distinct to Nigeria. When first identified, these IBADAN strains formed location clusters found in south-western Nigeria. It is possible that the geographical range of this “south-western IBADAN strain” has expanded to the north, supported by our data which revealed a closely related isolate in the northern state of Kano. However, it is also possible that there was also a “northern Nigeria” cluster (19), and that the isolate sequenced in our study may in fact be more closely related to these strains. There is limited information regarding the pathogenicity of this new IBADAN genotype, or effectiveness of vaccines against it, however, as yet, it has not been associated with clinical disease (17, 20). In our study, clinical disease was not reported in the flock where this sequence was obtained, and the farmer had reported vaccination with the polyvalent Izovac killed vaccine (ND + IB + egg drop syndrome, containing IBV strain Massachusetts M41). Antibody titers in the sampled birds in this flock were poor, which in addition to antigenic differences to the vaccine strain, may help explain why these birds were infected with this potentially avirulent variant, despite vaccination. Interestingly, IBV RNA was detected on 15 of 21 farms that had reported vaccinating against IBV at least once. As sequences could not be obtained from these isolates, and some could not be typed, it is unknown which strain these were and whether they were vaccine derived, or the results of vaccine failure.

Vaccination failure has previously been reported for IBV, and may have many causes, for example problems with formulation, storage, administration (55, 56), and, as discussed above, antigenic differences between vaccine and circulating strains resulting in incomplete protection (57–59). A vaccine may perform well in experimental studies, providing protection against either a homologous or heterologous challenge virus. However, these studies have important limitations, especially where specific-pathogen-free birds are utilized for experimental studies that may not represent the immune history of birds being vaccinated in the field, for example where maternally derived antibodies may interfere with the immune response (57, 58). Additionally, the thermo-instability of many vaccines may mean that a lack of cold chain storage may lead to degradation of vaccine components (60, 61). Indeed, problems with the maintenance of a cold chain for vaccine storage, and vaccines purchased a long distance from the farm were challenges reported by farmers as part of this project (Ekiri et al., in preparation). Incorrect reconstitution, dilution, timing and administration may also reduce the efficacy of a vaccine. Thus, studies evaluating the performance of vaccines in the field are crucial and are needed to help identify the cause of potential vaccine failures. Results from such studies, as well as further studies investigating circulating strains, may inform appropriate vaccine use in the field and future vaccine development. Additionally, findings from this study suggest there is a need for poultry farmer training and extension services focused on the importance of poultry vaccination, as well as appropriate vaccine handling and correct administration.

### Study Limitations

Although the data collected on some parameters reflects what was reported by farmers, it is possible that these data may have been biased or inaccurate, for example data on clinical records, time since last vaccine, records of specific vaccination (against NDV, IBDV, IBV), type of vaccine (live or killed vaccine), number of vaccine doses received, and vaccine strain administered. Where data was not available, clinical, or vaccine records were assumed to be absent, however, it is likely that some data may be incorrect based on observations of antibody titers, as discussed previously. Clinical and vaccine record data were collected through recall or use of farm records where available, and some data were either incomplete or missing. It is also possible that some of the information collected was prone to recall bias; for example, some of the farmers did not have a record of vaccines administered previously or records were incomplete or unavailable and therefore had to rely on memory alone. Incomplete data may have also impacted upon the conclusions made regarding whether a flock was protected against a particular disease. In addition, the authors recognize the definitions used for protection were not perfect and were prone to weaknesses/subjectivity.

In addition to data on clinical records, post-mortem examinations were performed on sick/dead birds. However, data regarding post-mortem lesions were not systematically collected, which would have been useful in the clinical diagnosis of disease present on the study farms, together with RT-PCR data. Further typing rRT-PCR data, or more sequences could not be obtained from positive samples for each of the three pathogens. For some samples, sequences obtained were not of sufficient quality, and for other samples, *C*_*T*_-values were high (>30) and therefore sequencing was not attempted due to the lower likelihood of returning a quality sequence.

Serum and tissue samples were collected from only 18 live birds and 2 sick/dead birds per farm, respectively. Study flock sizes varied from 100 to 16,088 birds and keeping sample sizes consistent over this large range may have biased results, and therefore results may be less representative of larger flocks. However, these sample sizes were considered appropriate given the budget and resources available for the project, and the authors feel that given the assumed biases, the results still provide useful information on which to build upon in further studies. Additionally, this study was limited to farms in only two states of Nigeria based on the study design and available budget resources. Oyo and Kano states were chosen as they were considered to be representative of states with large commercial poultry production in the south and north regions of the country, respectively. Although, the results from this study may also be relevant to other states, geographical differences were observed in this study, and therefore different strains may be circulating, or vaccination practices may differ, in different areas of Nigeria. Consequently, further studies should be conducted to investigate these factors in other states.

## Conclusions

This study assessed the level of protection according to antibody titers and identified the circulating strains of NDV, IBDV, and IBV on commercial poultry farms in Kano and Oyo States. Almost all the 44 study flocks did not have adequate protection against IB, and many did not have adequate protection against ND (despite vaccination) at the time of sampling, although more farms were considered to have adequate protection against IBD. Consequently, study findings suggested that many of the birds were at risk of field challenge to IBV and NDV. Additionally, IBDV and IBV RNA were detected on farms with a record of vaccination suggesting potential apparent vaccination failure or mismatch of vaccine and circulating strains. Studies evaluating the performance of vaccines in the field and those investigating the circulating strains are crucial to inform appropriate vaccine use in the field and future vaccine development. Such investigations could be supported with government funding for reference laboratories, to enable rapid detection and molecular epidemiological tracing. Adoption of appropriate vaccination programs should also be carefully considered by veterinarians and farmers based on the known circulating strains in a region, combined with sero-monitoring.

Finally, considering the wide and varying distribution of inadequate antibody titer protection especially against IBV and NDV, and the detection of field and vaccine strains on poultry farms in both Kano and Oyo states, it is important to consider training and education of farmers and poultry veterinarians on topics relevant to vaccination, such as designing appropriate vaccination programs, how to conduct and benefit from sero-monitoring, appropriate vaccine handling, storage, and application, and how to minimize the risks of vaccine failure. Indeed, as part of this study, specific recommendations were provided to each farm which included working with a poultry veterinarian to develop and revise the vaccination programmes based on known circulating strains, and to sero-monitor the flocks to assess the effectiveness of the administered vaccines. Putting these recommendations into effect, alongside those mentioned above may reduce the burden of these diseases and subsequently help improve poultry productivity and food security. It is important however, to consider the potential impact of challenges related to vaccine distribution and access to farmers and veterinarians especially in geographically distant regions.

## Data Availability Statement

The original contributions presented in the study are included in the article/Supplementary Material, further inquiries can be directed to the corresponding author/s.

## Ethics Statement

The studies involving human and animal participants were reviewed and approved by Ahmadu Bello University, Nigeria, Ethical Committee (Ethics approval number: ABUCAUC/2018/055) and the University of Surrey, United Kingdom, Animal Welfare and Ethical Review Board (NASPA Reference: NERA-1819-003). The participants provided their written informed consent to participate in this study. Written informed consent was obtained from the owners for the participation of their animals in this study.

## Author Contributions

AE and EM contributed to conception. AE, KA, EM, HG, B-VM, AW, and AC participated in the design of the study. AE, IE, EG, KA, RA, AO, OB, B-VM, and AW were involved in field implementation and data collection. AE, BA, KA, AW, IE, and EG were involved in data analysis and interpretation. AE, BA, and MD were involved in preparation of the manuscript. All authors read and approved the final manuscript.

## Funding

This study was supported by the African Livestock Productivity and Health Advancement (ALPHA) Initiative, co-funded by the Bill and Melinda Gates Foundation (BMGF) and Zoetis. Funding from Zoetis was an unrestricted grant. BMGF Grant number: OPP1165393.

## Author Disclaimer

The views and opinions expressed in this article are those of the authors and do not necessarily reflect the policy or position of any affiliated agency or institution of the authors.

## Conflict of Interest

This study was supported by the Africa Livestock Productivity and Health Advancement (ALPHA) Initiative, co-funded by Zoetis and the Bill and Melinda Gates Foundation. Funding from Zoetis was an unrestricted grant. BMGF Grant number: OPP1165393. Five of the co-authors (KA, EM, GV, AO, and OB) are employed by Zoetis and contributed to conception, implementation, and report writing. The remaining authors declare that the research was conducted in the absence of any commercial or financial relationships that could be construed as a potential conflict of interest.

## Publisher's Note

All claims expressed in this article are solely those of the authors and do not necessarily represent those of their affiliated organizations, or those of the publisher, the editors and the reviewers. Any product that may be evaluated in this article, or claim that may be made by its manufacturer, is not guaranteed or endorsed by the publisher.

## Footnotes

^1^Oliveros, J. C. *An Interactive Tool for Comparing Lists With Venn's Diagrams*. Available onine at: https://bioinfogp.cnb.csic.es/tools/venny/index.html.

## Abbreviations

GMT: geometric mean antibody titer
CV: coefficient of variation
IB: avian infectious bronchitis
IBD: infectious bursal disease
IBDV: infectious bursal disease virus
IBV: avian infectious bronchitis virus
ND: Newcastle disease
NDV: Newcastle disease virus
nv: non-virulent
PMV-1: paramyxovirus-1
vv: very virulent
C_T_: cycle threshold.
