# Supplementary material for: Evaluating Disease Threats to Sustainable Poultry Production in Africa: Newcastle Disease, Infectious Bursal Disease, and Avian Infectious Bronchitis in Commercial Poultry Flocks in Kano and Oyo States, Nigeria
Source: Front Vet Sci. 2021 Sep 8;8:730159. doi: 10.3389/fvets.2021.730159 (PMC8477209; doi:10.3389/fvets.2021.730159)
Supplement: Supplementary file 2 [file Data_Sheet_1.PDF]

Supplementary table 1. Real-time RT-PCR kits used for molecular testing of Newcastle disease virus, infectious bursal disease virus and infectious bronchitis virus.

Pathogen	rRT-PCR assay type	rRT-PCR Kit
Newcastle disease virus (NDV)	Serotype-specific	Kylt® Paramyxovirus 1 Real-Time RT-PCR Detection kit
Infectious bursal disease virus (IBDV)	Screen	Kylt® IBDV Screening Real-Time RT-PCR kit
	Serotype-specific	Kylt® IBDV Serotype 1 Pathotyping Real-Time RT-PCR kit
Infectious bronchitis virus (IBV)	Screen (species-specific)	Kylt® IB-aCoV kit
	Variant-specific	Kylt® IBV-Variant 02 Real-Time RT-PCR kit
	Variant-specific	Kylt® IBV-Variant 4/91 Real-Time RT-PCR kit
	Variant-specific	Kylt® IBV-Variant Arkansas Real-Time RT-PCR kit
	Variant-specific	Kylt® IBV-Variant D1466 Real-Time RT-PCR kit
	Variant-specific	Kylt® IBV-Variant D274 Real-Time RT-PCR kit
	Variant-specific	Kylt® IBV-Variant IB80 Real-Time RT-PCR kit
	Variant-specific	Kylt® IBV-Variant ItalyO2 Real-Time RT-PCR kit
	Variant-specific	Kylt® IBV-Variant Massachusetts Real-Time RT-PCR kit
	Variant-specific	Kylt® IBV-Variant Q1 Real-Time RT-PCR kit
	Variant-specific	Kylt® IBV-Variant QX Real-Time RT-PCR kit

Supplementary table 2. Frequency distribution of age, flock size, mortality and clinical signs reported on study farms.

		**State**		**Bird Type**			**Total**
**Variable**	**Response**	**Kano (%) (n=28)**	**Oyo (%) (n=16)**	**Layer (%) (n=33)**	**Broiler (%) (n=9)**	**Broiler-breeder (%) (n=2)**	**(%) (n= 44)**
Age category	2 - <4 weeks	0	2 (12.5)	0	2 (22.2)	0	2 (4.5)
	4 - <8 weeks	10 (35.7)	0	3 (9.1)	7 (77.8)	0	10 (22.7)
	8 - <20 weeks	3 (10.7)	2 (12.5)	4 (12.1)	0	1 (50.0)	5 (11.4)
	20 - <52 weeks	9 (32.1)	8 (50.0)	17 (51.5)	0	0	17 (38.6)
	>=52 weeks	4 (14.3)	4 (25.0)	7 (21.2)	0	1 (50.0)	8 (18.2)
	Not specified	2 (7.1)	0	2 (6.1)	0	0	2 (4.5)
Flock size	<200	1 (3.6)	0	0	1 (9.1)	0	1 (2.3)
	200 - <500	7 (25.0)	0	4 (12.1)	3 (33.3)	0	7 (15.9)
	500 - <2000	9 (32.1)	1 (6.3)	7 (21.2)	3 (33.3)	0	10 (22.7)
	2000 - <5000	3 (10.7)	9 (56.3)	10 (30.3)	0	2 (100.0)	12 (27.3)
	5000 - <15000	7 (25.0)	6 (37.5)	11 (33.3)	2 (22.2)	0	13 (29.5)
	>=15000	1 (3.6)	0	1 (3.0)	0	0	1 (2.3)
Mortality/week	<5	14 (50.0)	9 (56.3)	19 (57.6)	4 (44.4)	0	23 (52.3)
	5 - 9	5 (17.9)	3 (18.8)	5 (15.2)	3 (33.3)	0	8 (18.2)
	10 - 19	1 (3.6)	2 (12.5)	2 (6.1)	0	1 (50.0)	3 (6.8)
	20 - 49	5 (17.9)	2 (12.5)	5 (15.2)	1(11.1)	1 (50.0)	7 (15.9)
	50 - 99	1 (3.6)	0	0	1 (11.1)	0	1 (2.3)
	>=100	2 (7.1)	0	2 (6.1)	0	0	2 (4.5)
Clinical history[a]	Drop in production	0	3 (18.8)	1 (3.0)	0	2 (100.0)	3 (6.8)
	Respiratory distress	4 (14.3)	0	2 (6.1)	2 (22.2)	0	4 (9.1)
	Heat stress	5 (17.9)	0	5 (15.2)	0	0	5 (11.4)
	Stunted/slow growth	1 (3.6)	1 (6.3)	2 (6.1)	0	0	2 (4.5)
	Diarrhoea	2 (7.1)	2 (12.5)	1 (3.0)	2 (22.2)	1 (50.0)	4 (9.1)
	Sudden deaths	0	2 (12.5)	0	0	2 (100.0)	2 (4.5)
	None reported	8 (28.6)	4 (25.0)	10 (30.3)	2 (22.2)	0	12 (27.3)

[a]Five farms in Oyo did not provide details of recent clinical history

Supplementary table 3. Flock vaccination history.

Variable	Response	State		Bird Type			Total
		**Kano (%) (n=28)**	**Oyo (%) (n=16)**	**Layer (%) (n=33)**	**Broiler (%) (n=9)**	**Broiler breeder (%) (n=2)**	**(%) (n= 44)**
Farms vaccinated against NDV?	Yes	28 (100.0)	16 (100.0)	33 (100.0)	9 (100.0)	2 (100.0)	44 (100.0)
	No	0	0	0	0	0	0
Number of NDV vaccine doses	1	1 (3.6)	16 (100.0)	12 (36.4)	3 (33.3)	2 (100.0)	17 (38.6)
	2	7 (25.0)	0	1 (3.0)	6 (66.7)	0	7 (15.9)
	3	13 (46.4)	0	13 (39.4)	0	0	13 (29.5)
	4	7 (25.0)	0	7 (21.2)	0	0	7 (15.9)
NDV vaccine strain given[a]	Komorov	5 (17.9)	3 (18.8)	5 (15.2)	1 (11.1)	2 (100.0)	8 (18.2)
	Unspecified mesogenic	0	5 (31.3)	4 (12.1)	1 (11.1)	0	5 (11.4)
	R2B	0	3 (18.8)	2 (6.1)	1 (11.1)	0	3 (6.8)
	LaSota	23 (82.1)	3 (18.8)	20 (60.6)	6 (66.7)		26 (59.1)
	B1	6 (21.4)	0	6 (18.2)	0	0	6 (13.6)
	VH	5 (17.9)	1 (6.25)	5 (15.2)	1 (11.1)	0	6 (13.6)
Farms vaccinated against IBDV?	Yes	28 (100.0)	4 (25.0)	23 (69.7)	9 (100.0)	0	32 (72.7)
	No	0	12 (75.0)	10 (30.3)	0	2 (100.0)	12 (27.3)
Number of IBDV vaccine doses	0	0	12 (75.0)	10 (30.3)	0	2 (100.0)	12 (27.3)
	1	0	4 (25.0)	2 (6.1)	2 (22.2)	0	4 (9.1)
	2	28 (100.0)	0	21 (63.6)	7 (77.8)	0	28 (63.6)
IBDV vaccine strain given[a]	Intermediate	15 (53.6)	0	12 (36.4)	3 (33.3)	0	15 (34.1)
	Intermediate Plus	4 (14.3)	4 (25.0)	6 (18.2)	2 (22.2)	0	8 (18.2)
	Virgo 7	7 (25.0)	0	4 (12.1)	3 (33.3)	0	7 (15.9)
	Not specified	2 (7.1)	0	1 (3.0)	1 (11.1)	0	2 (4.5)
Number of IBV vaccine doses	0	21 (75.0)	2 (12.5)	15 (45.5)	8 (88.9)	0	23 (52.3)
	1	7 (25.0)	14 (87.5)	18 (54.5)	1 (11.1)	2 (100.0)	21 (47.7)
IBV vaccine strain given[a]	H120	4 (14.3)	4 (25.0)	7 (21.2)	1 (11.1)	0	8 (18.2)
	D274 clone	0	1 (6.25)	1 (3.0)	0	0	1 (2.3)
	Not specified	3 (10.7)	10 (62.5)	11 (33.3)	0	2 (100.0)	13 (29.5)

[a]Vaccine strain given was as reported/specified by the farmer. More than one vaccine strain may have been administered on one farm.

Supplementary Figure Legends

Supplementary figure 1. Real-time RT-PCR results for NDV (A), IBDV (B) and IBV (C), by sample type and viral strain. For IBDV (B) all samples were bursal tissue, and for IBV (C) samples types were tracheal and oropharyngeal swab ([a]) and cecal tonsil ([b]).
