# Supplementary figures and images for: Evaluating Disease Threats to Sustainable Poultry Production in Africa: Newcastle Disease, Infectious Bursal Disease, and Avian Infectious Bronchitis in Commercial Poultry Flocks in Kano and Oyo States, Nigeria
Source: Front Vet Sci. 2021 Sep 8;8:730159. doi: 10.3389/fvets.2021.730159 (PMC8477209; doi:10.3389/fvets.2021.730159)

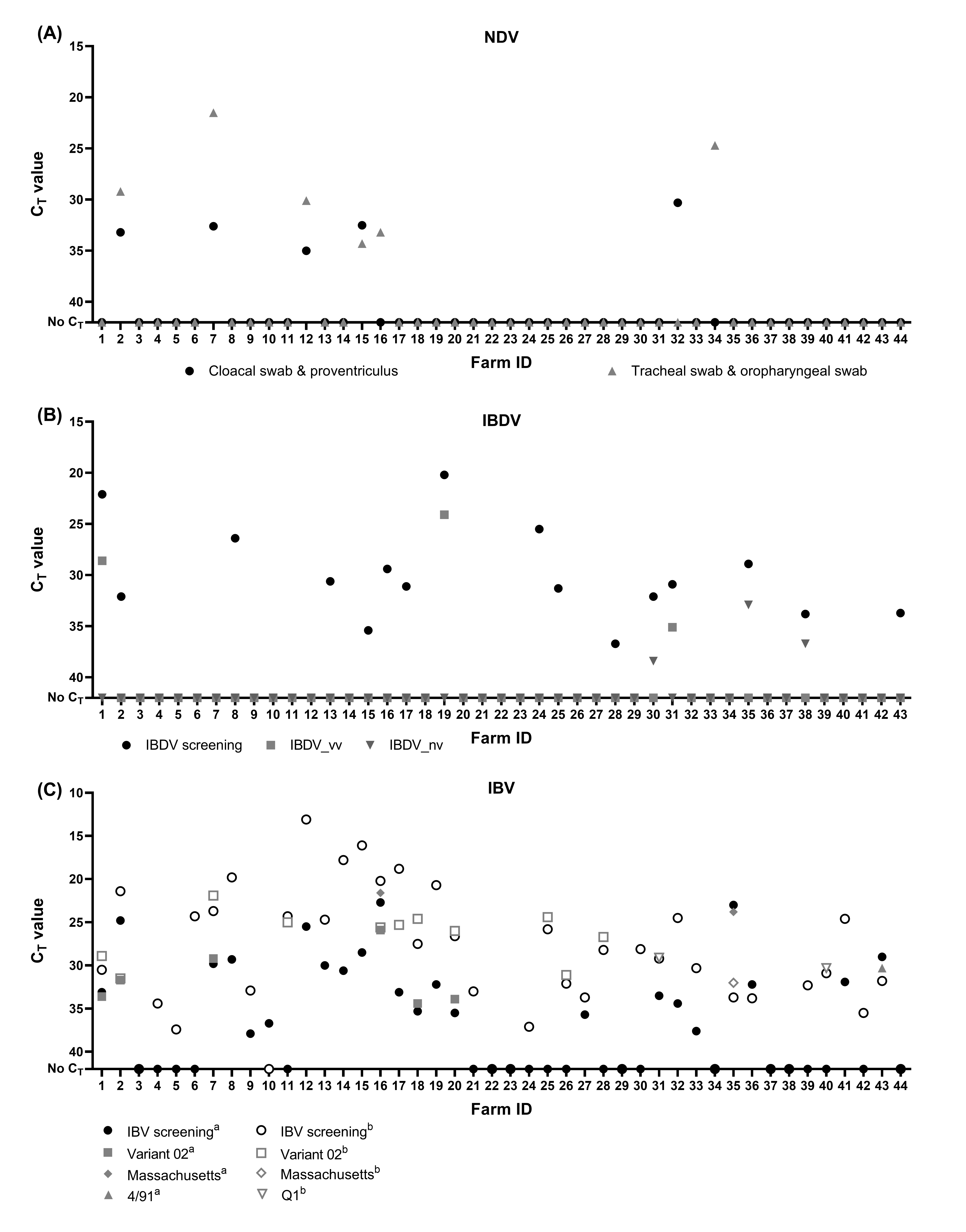

Supplement: Supplementary file 3 [file Image_1.TIF]
